# Improving visual functions in adult amblyopia with combined perceptual training and transcranial random noise stimulation (tRNS): a pilot study

**DOI:** 10.3389/fpsyg.2014.01402

**Published:** 2014-12-09

**Authors:** Gianluca Campana, Rebecca Camilleri, Andrea Pavan, Antonella Veronese, Giuseppe Lo Giudice

**Affiliations:** ^1^Department of General Psychology, University of PadovaPadova, Italy; ^2^Human Inspired Technologies Research Centre, University of PadovaPadova, Italy; ^3^Institut für Experimentelle Psychologie, Universität RegensburgRegensburg, Germany; ^4^San Paolo Ophthalmic Center, San Antonio HospitalPadova, Italy

**Keywords:** amblyopia, visual acuity, contrast sensitivity, perceptual learning, lateral masking, tRNS

## Abstract

Amblyopia is a visual disorder due to an abnormal pattern of functional connectivity of the visual cortex and characterized by several visual deficits of spatial vision including impairments of visual acuity (VA) and of the contrast sensitivity function (CSF). Despite being a developmental disorder caused by reduced visual stimulation during early life (critical period), several studies have shown that extensive visual perceptual training can improve VA and CSF in people with amblyopia even in adulthood. With the present study we assessed whether a much shorter perceptual training regime, in association with high-frequency transcranial electrical stimulation (hf-tRNS), was able to improve visual functions in a group of adult participants with amblyopia. Results show that, in comparison with previous studies where a large number sessions with a similar training regime were used (Polat et al., 2004), here just eight sessions of training in contrast detection under lateral masking conditions combined with hf-tRNS, were able to substantially improve VA and CSF in adults with amblyopia.

## INTRODUCTION

Amblyopia, sometimes referred to as “lazy eye,” is a developmental visual disorder characterized by several functional impairments in spatial vision (even with the best optical correction) in absence of any organic defects of the eye besides the refractive ones (Ciuffreda et al., 1991; McKee et al., 2003; Robaei et al., 2006). Impairments range from a reduction of visual acuity (VA), contrast sensitivity function (CSF) and Vernier acuity, to abnormal spatial interactions (Polat et al., 1997; Levi et al., 2002) or deficiencies in stereopsis (Wallace et al., 2011). It is believed to be due to an anomalous pattern of functional connectivity within the primary visual cortex, in particular of neurons selective for orientation and spatial frequency (Polat, 1999), thus causing abnormal processing of visual information coming from one or both eyes (but typically only one eye is involved). Until recently, amblyopia was thought to be untreatable after the “critical period” spanning up to the first decade of life (Epelbaum et al., 1993; Greenwald and Parks, 1999; Loudon et al., 2002), due to diminished neural plasticity within the visual cortex that would limit any anatomical, physiological or functional changes (Berardi et al., 2003).

Numerous studies, however, have reported large and stimulus-specific performance improvements (perceptual learning) in normal adults following training in various visual tasks (Fiorentini and Berardi, 1980; Karni and Sagi, 1991; Poggio et al., 1992; Schoups et al., 1995; see Sagi, 2011 for a review), pointing to neuronal plasticity at early levels of the adult visual system (Schoups et al., 2001; Pourtois et al., 2008). In fact, over the past 15 years, marked improvements of various visual functions in adults with amblyopia, following extensive sessions of perceptual learning, have been reported (see Levi and Li, 2009 and Polat, 2009; Astle et al., 2011a,b for recent reviews). Different authors used different training tasks, ranging from Vernier acuity (Levi and Polat, 1996; Levi et al., 1997), stereo acuity (Astle et al., 2011a), to position discrimination in noise (Li and Levi, 2004; Li et al., 2005, 2007), identification of luminance-defined letters in noise (Levi, 2005) or contrast-defined letters (Chung et al., 2006, 2008), contrast detection, either with Gabor stimuli or letters in isolation (Zhou et al., 2006; Huang et al., 2008; Astle et al., 2011c), or when Gabors were flanked by similar collinear patches (i.e., lateral masking; Polat et al., 2004). Analysing the amount of improvement as a function of the task used in different studies, Levi and Li (2009) pointed out that in most studies the ratio of improvement between post- and pre-training contrast sensitivity (CS) thresholds is between 0.4 and 0.8 for both VA and CSF. The task that obtained the largest improvement ratio on both measurements (∼0.35) was a contrast detection task using the lateral masking procedure (Polat et al., 2004). Focusing on the abnormal spatial interactions in amblyopia, Polat et al. (2004) used a training procedure that allowed a strengthening of facilitatory lateral interactions and a weakening of inhibitory lateral interactions between detectors tuned to specific orientations and spatial frequencies, thus obtaining a large and consistent improvement in VA (78% gain, equal to 0.25 LogMAR improvement) and CSF (improvement ranging from 2.05 to 4.23 times) in adults with amblyopia.

A drawback of this and similar training paradigms, however, is their duration: the large number of sessions required to achieve such improvements (from 30 to 80 sessions) could either prevent amblyopic patients from starting the training or lead to a high number of dropouts.

Recent studies have pointed out how non-invasive transcranial brain stimulation techniques are able to boost perceptual learning in normal observers. In particular, it has been shown that online transcranial electrical stimulation using random frequencies in the high-frequency range (high-frequency transcranial random noise stimulation, hf-tRNS), is the most efficacious type of electrical stimulation for enhancing and accelerating within-session perceptual learning (Fertonani et al., 2011; Pirulli et al., 2013).

In this study we assessed the extent of VA and CSF improvement in a small sample (*N* = 7) of patients with anisometric amblyopia, following a brief training (eight sessions) in contrast detection of a central Gabor patch (target) flanked by two high contrast Gabor patches of the same spatial frequency (i.e., lateral masking; Polat et al., 2004), in conjunction with online hf-tRNS.

## MATERIALS AND METHODS

### PARTICIPANTS

Seven participants with anisometric amblyopia were recruited at the San Paolo Ophthalmic Center of San Antonio Hospital (Padova, Italy) during routine ophthalmological assessment (mean age of 39.20, ranging between 26 and 52). The participants were enrolled in a 2-week (eight sessions) behavioral training program using a contrast detection task under lateral masking conditions (Polat et al., 2004; Polat, 2009) combined with online high frequency tRNS (hf-tRNS).

All pre/post tests were administered monocularly on either eye and with the best optical correction. Perceptual training was also administered monocularly on the amblyopic eye with the best optical correction. Exclusion criteria included any other ocular condition or cause for reduced VA other than amblyopia, myopia, presbyopia, hypermetropia and/or astigmatism; these include diabetes mellitus, pregnancy, presence of myopia-related ocular complications and any previous ocular surgery. Exclusion criteria also included incompatibility with transcranial electrical stimulation, as assessed with a questionnaire (e.g., history of seizures, skin problems, migraine, etc.). This study has been approved by the local Ethics Committee.

### EXPERIMENTAL PROCEDURE

Before (pre-tests) and after the training (with tRNS; post-tests), VA and CSF were assessed for each participant by using, respectively, Landolt C of the Freiburg Visual Acuity Test (FrACT, Bach, 1996), and the CRS Psycho 2.36 test (Cambridge Research Systems Ltd, Rochester, UK) from a viewing distance of 1.5 m.

Visual acuity was measured with an orientation discrimination task (eight possible orientations of the gap of the Landolt C). The Best-Pest adaptive procedure was used to calculate the threshold corresponding to 62.5% of correct discrimination. Stimulus duration lasted until the participants’ response. An auditory cue was presented upon stimulus presentation and a different auditory cue was used as feedback for incorrect responses.

Contrast sensitivity was measured with the method of adjustment by asking the participant to adjust the contrast of a vertical sinusoidal grating covering the whole screen (21.3 × 16°), with four ascending (from lower to higher grating contrast) and four descending (from higher to lower grating contrast) series. The initial contrast on the first descending series was set according to pilot experiments, ranging from -15 dB (17.78% contrast) at intermediate spatial frequencies, to 0 dB (100% contrast) at high spatial frequencies. On successive series the starting contrast for each tested spatial frequency was set as the contrast threshold obtained in the previous series, plus (in descending series) or minus (in ascending series) a factor between 6 and 10 dB (randomly selected). Increments/decrements were equal to 1 dB. The resulting contrast threshold was the arithmetic mean of the last selected contrast for each of the eight series, independently for each spatial frequency. Each tested spatial frequency (ranging from 0.8 to 14.5 cpd) was presented sequentially starting from the lower spatial frequency and progressively moving on to the higher spatial frequencies; five different spatial frequencies were tested. For each participant, CS at each tested spatial frequency was calculated by averaging across series.

**FIGURE 1:**
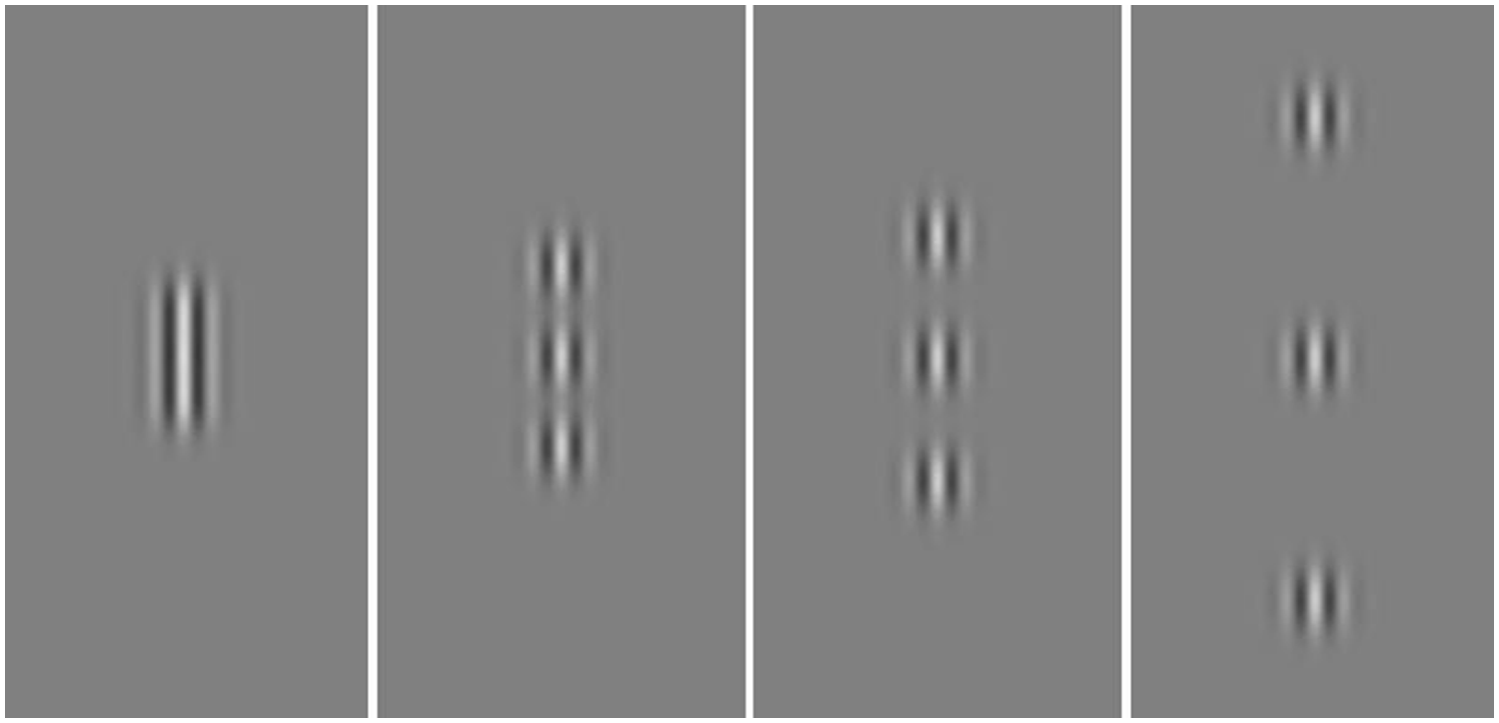
**Example of stimuli used in the training.** The central Gabor was the target varying in contrast according to a staircase. Flanking Gabors had a fixed contrast of 0.6 Michelson contrast. The target-to-flankers distance was varied across blocks (i.e., 1.5, 3, 4, and 8λ; from left to right). The contrast of the target was increased for demonstrative purposes.

The behavioral training consisted of a two-interval forced choice (2IFC) task where the participants had to detect the presence of a central Gabor, which changed in contrast according to the performance of the participant, surrounded by two high-contrast (0.6 Michelson contrast) collinear Gabors (flankers; **Figure 1**). Gabors were made of a cosinusoidal carrier enveloped by a stationary Gaussian. Standard deviation of the luminance Gaussian envelope (σ) was equal to the sinusoidal wavelength (λ); that is, the size of the Gabor patches covaried with their spatial frequency. Additionally, the spatial phase of the cosinusoidal carrier equalled to zero (evenly symmetric Gabor patch). Center-to-center distance between target and flankers was varied across blocks (1.5, 3, 4, and 8λ). On each session two blocks were administered with the same center-to-center distance. The order of presentation always started with the largest distance and ended with the smallest distance. Stimulus duration lasted 200 ms. Contrast threshold, corresponding to 79% of correct responses, was determined by using a 1up/3down staircase procedure on the last eight reversals (Levitt, 1971). Participants underwent eight training sessions during 2 weeks (four consecutive sessions per week), and trained on four different orientations of the stimulus (that changed every 2 days) with a single spatial frequency, chosen according to the individual’s cut-off performance in the pre-test CS measurement, defined as the spatial frequency at which the estimated contrast threshold from pre-training CS measurements was 0.50 (Michelson contrast; Zhou et al., 2006). Trained spatial frequencies ranged from 3 to 15 cpd. Each session consisted of eight blocks each containing 60 trials, which lasted for approximately 45 min. The total training time for each participant, across the 2 weeks was approximately 6 h.

Participants were administered hf-tRNS (1.5 mA) during the first five blocks on each session (Fertonani et al., 2011). In order to reduce spatial and temporal uncertainty both an auditory and a spatial cue were implemented. On each trial a central fixation point preceded the presentation of each interval. Performance feedback was also provided to the participants in the form of an auditory beep following an incorrect response.

The main differences between the training procedure used in the present study with respect to that of Polat et al. (2004), besides the use of online tRNS and a smaller number of sessions, are: the use of a range of durations (80–320 ms) vs. a fixed duration (200 ms) for stimulus presentation in our study; the alternate use of target with or without flankers vs. a constant use of flankers in our study; the use of an automated and computerized decision-maker algorithm for deciding the parameters (spatial frequency, orientation) to be used in subsequent sessions vs. a relatively fixed sequence of parameters in our study.

### APPARATUS

Training and VA tests were displayed on a 22-inch Philips Brilliance 202P4 monitor with a refresh rate of 60 Hz and a resolution of 1280 × 1024 pixels. The monitor was luminance-calibrated (gamma-corrected with γ = 1). The stimuli used in the training were generated with the Matlab Psychtoolbox (Brainard, 1997; Pelli, 1997), whereas stimuli for measuring VA were generated using the Freiburg Acuity and Contrast Test (FrACT 3.8, Bach, 1996). All stimuli were presented centrally. Viewing distance was equal to 3 m for VA tests, whereas the training was administered from 1.5 m. Background screen luminance (corresponding to mean luminance of Gabor stimuli) was 31.5 cd/m^2^.

Contrast sensitivity tests were displayed on a 17-inch CRT monitor (Brilliance 107P; Philips) with a refresh rate of 70 Hz and a resolution of 1024 × 768 pixels. The monitor was luminance-calibrates with γ = 1. The stimuli were generated with the CRS Psycho 2.36 test (CRS Psycho 2.36; Cambridge Research Systems Ltd, Rochester, UK) on a computer equipped with a 12-bit resolution graphics card (Cambridge Research Systems Ltd VSG2/3). Viewing distance was equal to 1.5 m. Background screen luminance (corresponding to mean luminance of the gratings) was 48.5 cd/m^2^. All tests and the training were carried out in a dark and silent room.

### tRNS

High frequency transcranial random noise stimulation was delivered using a battery-driven stimulator (BrainSTIM, EMS) through a pair of saline-soaked sponge electrodes. The tRNS consisted of an alternating current of 1.5 mA intensity with a 0 mA offset applied at random frequencies. The frequencies ranged from 100 to 640 Hz.

The stimulations were applied for approximately 5 min (equalling the duration of a training block) during each of the first five training blocks (Fertonani et al., 2011); thus, the total duration of the stimulation was ∼25 min. This stimulation protocol has been demonstrated efficacious in boosting perceptual learning in previous studies (Fertonani et al., 2011; Pirulli et al., 2013). The active electrode had an area of 16 cm^2^ and was placed over the occipital cortex measured at ∼3 cm above the inion. The reference electrode had an area of 60 cm^2^ and was placed on the forehead. The current density was maintained well below the safety limits (always below 1 A/m^2^; Poreisz et al., 2007). The electrodes were kept in place with bandages.

## RESULTS

**FIGURE 2:**
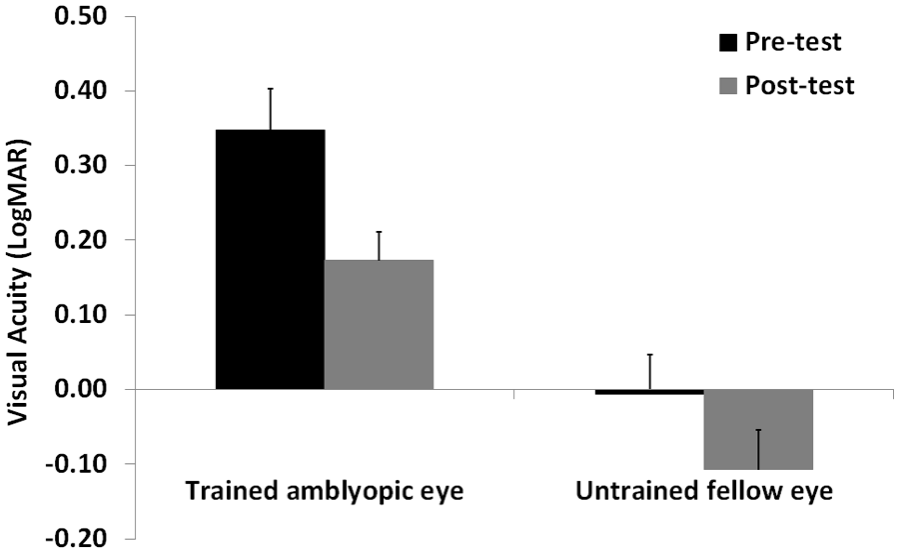
**Mean visual acuity before (Pre-test) and after (Post-test) the lateral masking training with concurrent hf-tRNS, separately for the trained amblyopic eye and the untrained fellow eye.** Error bars represent 1 SEM.

Visual acuity and CS data were analyzed with a repeated measures ANOVA with Time (pre- vs. post-test), and Spatial Frequency (only for CS: 0.8, 2.9, 5.8, 9.7, and 14.5 cpd) as within-subjects factors, and Eye (amblyopic/trained vs. non-amblyopic/untrained) as a between-subjects factor. When data violated the assumption of sphericity, as assessed with the Mauchly’s test, we applied the Greenhouse-Geisser correction of the degrees of freedom. Following eight sessions of a contrast detection training with lateral masking, VA significantly improved in both trained and untrained eye (*F*_1,12_ = 35.4, *p* = 0.0001, $\eta_{p}^{2}$ = 0.75). The interaction between Training Time and Eye was not significant (*F*_1,12_ = 2.47, *p*= 0.14, $\eta_{p}^{2}$ = 0.17), indicating a similar improvement on both trained and untrained eyes. Overall mean improvement was equal to 0.14 LogMAR, with a mean improvement close to 2 LogMAR lines (0.18 LogMAR, corresponding to 50% improvement, that is from 0.35 LogMAR to 0.17 LogMAR), in the trained (amblyopic) eye, and equal to 0.1 LogMAR, that is from 0 LogMAR to -0.1 LogMAR in the untrained eye (**Figure 2**). The VA in the trained and untrained eye was also significantly different (*F*_1,8_ = 22.12, *p* = 0.001, $\eta_{p}^{2}$ = 0.65).

**FIGURE 3:**
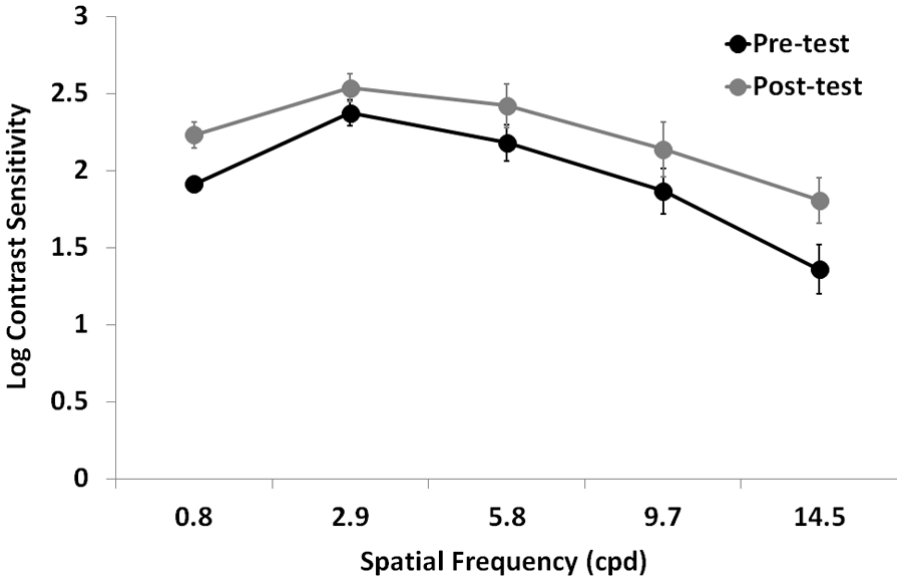
**Mean Log-CSF (average of the two eyes) before (Pre-test) and after (Post-test) the lateral masking training with concurrent hf-tRNS.** Error bars represent ± 1 SEM.

Contrast sensitivity significantly improved after training (*F*_1,12_ = 11.7, *p* = 0.005, $\eta_{p}^{2}$ = 0.49), regardless the eye (interaction Time by Eye: *F*_1,12_ = 0.03, *p* = 0.87, $\eta_{p}^{2}$ = 0.02; **Figure 3**). As expected, there was also a large CS variation across the different spatial frequencies tested (*F*_1.5,18_ = 29.7, *p* = 0.0001, $\eta_{p}^{2}$ = 0.71), a significant difference in CS between the two eyes (*F*_1,12_ = 8.8, *p* = 0.012, $\eta_{p}^{2}$ = 0.42), and a significant interaction Time by Spatial Frequency (*F*_4,48_ = 2.7, *p* = 0.043, $\eta_{p}^{2}$ = 0.18), suggesting that the CS improvement could have occurred only at certain spatial frequencies.

In order to test this hypothesis, we performed further analysis separately for each spatial frequency. Repeated-measures ANOVA with Training Time (pre- vs. post-test) as a within-subject factor, and Eye (trained vs. untrained) as a between-subjects factor showed a significant difference between pre- and post-test at all tested spatial frequencies (0.8 cpd: *F*_1,12_ = 7, *p* = 0.021, $\eta_{p}^{2}$ = 0.37; 2.9 cpd: *F*_1,12_ = 11.2, *p* = 0.006, $\eta_{p}^{2}$ = 0.48; 5.8 cpd: *F*_1,12_ = 11.5, *p* = 0.005, $\eta_{p}^{2}$ = 0.49; 9.7 cpd: *F*_1,128_ = 5.8, *p* = 0.03, $\eta_{p}^{2}$ = 0.33; 14.5 cpd: *F*_1,12_ = 5.4, *p* = 0.04, $\eta_{p}^{2}$ = 0.31), regardless the eye (interaction Time by Eye was not significant in any of the tested spatial frequencies). In terms of percentage improvement with respect to pre-test, CS in the trained eye had nearly a twofold improvement (averaged across participants and spatial frequencies), ranging from 74% at the lowest tested spatial frequency to 435% at the highest tested spatial frequency, whereas CS in the untrained eye had a mean CS improvement of 60% (averaged across participants and spatial frequencies), ranging from 21% at intermediate spatial frequency (2.9 cpd) to 165% at the lowest tested spatial frequency.

## DISCUSSION

In our small sample of participants, a short (eight sessions) contrast detection training under lateral masking conditions and concurrent hf-tRNS was able to increase mean VA by 0.18 LogMAR (53% improvement, ranging from 25 to 111%) in the trained amblyopic eye. An improvement between 2 and 3 LogMAR lines was achieved in four participants out of seven. This could be considered a smaller improvement in comparison to the results obtained by Polat et al. (2004), where a similar training procedure was used, but with a training regime of 48 sessions on average (VA increased by 0.25 LogMAR, 78% improvement). However, if we compare our results with the improvement attained by Polat et al. (2004) after eight sessions (0.13 LogMAR, 35% improvement), and considering that in our study the mean best-corrected VA reached 0.18 LogMAR (better than 6/9, the upper limit for normal vision), we can state that a marked and clinically relevant improvement in VA was obtained in a relatively short time frame.

The CSF also resulted in strong improvements following training, both in the trained amblyopic eye and in the untrained fellow eye. CSF in the trained amblyopic eye increased at all tested spatial frequencies by a factor of 1.05, 0.74, 1.13, 1.35, and 3.21 for spatial frequencies of 0.8, 2.9, 5.8, 9.7, and 14.5 cpd, respectively. Compared with the results of Polat et al. (2004) obtained with 48 sessions of training (CSF improved by a factor of 2.21, 2.12, 2.93, 4.23, and 2.05 for spatial frequencies of 1.5, 3, 6, 12, and 18 cpd), we also see that for the CSF the improvement we estimated appears smaller, although obtained this with 1/6th of the total amount of sessions. Most importantly, the largest improvement in our participants was at a similar high spatial frequency (14.5 cpd) compared to the largest improvement obtained by Polat et al. (2004, 12 cpd), and with a relatively similar improvement factor (3.21 vs. 4.23). On the other hand, a smaller improvement (by a factor of 2.05) was found by Polat et al. (2004) at 18 cpd. Although it is still possible that the results obtained with the present study with respect to that of Polat et al. (2004) were due to the slightly different conditions used, besides the different training duration and the use of tRNS (e.g., different target durations, the use of a computerized decision-maker algorithm to decide the parameters to be used in subsequent sessions), we believe that the conditions used by Polat et al. (2004) could in fact be more efficient in producing perceptual learning and transfer to related and unrelated visual functions. For example, training lateral interactions with a variable and faster stimulus presentation has been shown to improve not just CS but also processing speed, thus increasing the “improvement of other functions that are processed either at the same or at later stages” (Polat, 2009), and the use of computerized decision-maker algorithms should always supply the participant with the most effective stimulation parameters.

Although the present study lacks a Sham group and therefore the effects of tRNS cannot be isolated, the underlying mechanisms as to how tRNS administered over the visual cortices could boost learning of CS that would transfer onto unrelated tasks such as VA. Being a sub-threshold stimulation, which is repetitive and random in nature, whilst engaged in a contrast detection task, tRNS could be inducing temporal summation of small depolarizing currents that interact with the concurrent activity of cortical neurons which are tuned to specific orientations and spatial frequencies, thus enhancing performance on the task and inducing synaptic potentiation (Fertonani et al., 2011). In fact, Pirulli et al. (2013) showed that perceptual learning in a visual discrimination task only improved when the hf-tRNS was administered during task execution (online stimulation), while no improvement was found when it was administered with no concurrent task (oﬄine).

Taken together these data suggest that a short perceptual training combined with online hf-tRNS can induce brain plasticity and can considerably improve visual functions in the amblyopic eye. Further studies are needed to confirm the present results on a larger sample of participants, and to estimate the best ratio between extent of improvements of visual functions and duration of the perceptual training combined with hf-tRNS. While the contribution of hf-tRNS on perceptual improvements has already been shown both in participants with normal sight (Fertonani et al., 2011; Pirulli et al., 2013) and in participants with uncorrected myopia (Camilleri et al., 2014), future studies with sham controls are needed to determine the precise contribution of hf-tRNS on such improvements in amblyopia.

## Conflict of Interest Statement

The authors declare that the research was conducted in the absence of any commercial or financial relationships that could be construed as a potential conflict of interest.
